# Prevalence and risk factors for depression among training physicians in China and the United States

**DOI:** 10.1038/s41598-022-12066-y

**Published:** 2022-05-17

**Authors:** Lihong Chen, Zhuo Zhao, Zhen Wang, Ying Zhou, Xin Zhou, Hui Pan, Fengtao Shen, Suhua Zeng, Xinhua Shao, Elena Frank, Srijan Sen, Weidong Li, Margit Burmeister

**Affiliations:** 1 Key Laboratory for the Genetics of Development and Neuropsychiatric Disorders (Ministry of Education), Shanghai Key Laboratory of Psychotic Disorders, Brain Science and Technology Research Center and Institute of Psychology and Behavioral Science, Shanghai Jiao Tong University, Life Science Building 1-209, 800 Dongchuan Road, Shanghai, 200240 China; 2 Michigan Neuroscience Institute, University of Michigan, Ann Arbor, USA; 3 Department of Hospital Administration, Shanghai Jiao Tong University School of Medicine, Shanghai, China; 4 Shanghai Mental Health Center, Shanghai Jiao Tong University School of Medicine, Shanghai, China; 5 Peking Union Medical College Hospital, Beijing, China; 6 Departments of Psychiatry and of Computational Medicine and Bioinformatics, University of Michigan, Ann Arbor, USA

**Keywords:** Human behaviour, Epidemiology, Health policy

## Abstract

During their first year of medical residency (internship), 35% of training physicians in the United States suffer at least one depression episode. We assessed whether there is a similar increase of depression among first year residents in China, and identified predictors of depression in the two systems. 1006 residents across three cohorts (2016–2017, 2017–2018 and 2018–2019) at Shanghai Jiao Tong University and Peking Union Medical College were assessed in parallel with three cohorts of 7028 residents at 100 + US institutions. The Patient Health Questionnaire-9 (PHQ-9) depressive symptoms were measured at baseline and quarterly. Demographic, personal and residency factors were assessed as potential predictors of PHQ-9 depression scores. Similar to training interns in the US, the proportion of participants in China who met depression criteria at least once during the first year of residency increased substantially, from 9.1 to 35.1%. History of depression and symptoms at baseline were common factors significantly associated with depression during residency. By contrast, neuroticism, early family environment, female gender and not being coupled were associated with depression risk only in the US, while young age was a predictor of depression only in China. Fear of workplace violence also was a predictor in China. Long duty hours and reduced sleep duration emerged as training predictors of depression in both countries. The magnitude of depression increase and work-related drivers of depression were similar between China and the US, suggesting a need for effective system reforms in both systems.

## Introduction

As a leading cause of disability worldwide, depression is a serious global health concern^1^. In China, increased attention has been paid to monitoring mental health in recent years, however data on prevalence and risk factors based on consistent, validated measures remain scarce. Preliminary evidence from the first nationally representative survey of mental health in China^2^ suggests the prevalence of mental disorders is increasing, and that depression is the most common mood disorder. As the world’s most populous country, developing a deeper understanding of the incidence and drivers of depression in China is critical to inform prevention efforts and reduce the overall global burden of disease.

With high rates of violence committed by patients and their families against healthcare workers^3–7^ in China, the mental health of physicians is of particular concern. In addition, the first year of physician residency training is known as one of the most stressful periods during a medical career, characterized by high workload, new responsibilities, and inconsistent and insufficient sleep^8–10^. A large meta-analysis including 54 studies across 16 other countries found that depression increases five-fold during the first year of training, with 25% to 30% of residents fulfilling the criteria for depression at any given time^3^. In China, recent data indicate high rates of anxiety and depressive symptoms among healthcare workers during the COVID-19 pandemic^11–13^, including first year residents^14^, however the prevalence of depression and associated risk factors among training physicians in China is otherwise not known.

In 2014, China introduced a standardized residency program^15,16^, providing an opportunity to systematically assess depression among residents in China. Given the ongoing COVID-19 risk world-wide and the crucial role of residents in fighting against the pandemic, it is of great importance to call for public attention on their mental health and reveal the driven factors of depression among them. As socioeconomic context and cultural norms can influence the epidemiology of depression, it is imperative to consider the commonalities and differences in drivers of depression between training physicians in the US and China to inform intervention efforts in both countries. Here, we utilize longitudinal data collected in parallel cohorts in the US and China using the well-established Intern Health Study protocol^10,17^ to determine the prevalence and magnitude of depressive symptoms prior to and during the first year of residency, and to identify baseline and within-residency predictors of depression among trainees in both countries.

## Methods

All methods were performed in accordance with the relevant guidelines and regulations.

### Participants

We assessed three cohorts of residents in China, enrolled between 2016 and 2018, in 16 hospitals in Shanghai and Beijing. Two weeks before start of residency, when the classes for each hospital are verified, an email invitation was sent to eligible first-year residents at participating hospitals. We invited 3666 first year residents, with 1664 (45%) agreeing to take part and completing the online baseline survey. 1006 of them finished at least one follow-up survey and were included in the analysis. In parallel, we assessed three cohorts of residents in the US, enrolled between 2016 and 2018, representing over 100 institutions across the country^18^. We invited 14,723 first year residents, with 8266 (56%) agreeing to take part and completing the online baseline survey online. 7028 of them completed at least one follow-up survey and were included in the analysis. All participants in both countries provided electronic informed consent. This study design and informed consent procedure was approved by the Ethics committees of Peking Union Medical College, the University of Michigan, and Shanghai Jiao Tong University.

#### Baseline assessment

Prior to commencing residency, participants completed an initial survey via secure website or mobile app. The survey assessed depressive symptoms using the 9-item Patient Health Questionnaire (PHQ-9). The PHQ-9 is a self-report component of the Primary Care Evaluation of Mental Disorders inventory, designed to screen for depressive symptoms^19^. For each of the nine depressive symptoms, residents indicated whether, during the previous two weeks, the symptom had bothered them “not at all,” “several days,” “more than half the days,” or “nearly every day.” Each item yields a score of 0 to 3, so that the PHQ-9 total score ranges from 0 to 27^19^. The PHQ-9 has been validated and used extensively in Chinese^20^. A PHQ-9 score of ≥ 10 has a sensitivity of 93% and a specificity of 88% for the diagnosis of major depressive disorder^21^.

We also assessed general demographic factors (age, sex, ethnicity and marital status, Table 1), medical education factors (medical institution and specialty), self-reported history of depression, and the following psychological measures: (1) neuroticism (part of the NEO-Five Factor Inventory)^22^, (2) early family environment (Risky Families Questionnaire)^23^. For first year residents in China, validated Chinese versions of inventories and translations of non-inventory items were administered.

**Table 1:** Sample Demographic Characteristics at Baseline.

Characteristic	Number (%)		*P* value
	China (n = 1006)	US (n = 7028)	China versus US
Age, mean (SD), year	25.0 ± 2.5	27.5 ± 2.6	< 0.001
Gender (female)	670 (66.6)	3662 (52.1)	< 0.001
**Specialty**			0.44
Internal medicine	207 (20.7)	1604 (22.8)	
General surgery	189 (18.9)	637 (9.1)	
Obstetrics/gynecology	57 (5.7)	457 (6.5)	
Pediatrics	89 (8.9)	941 (13.4)	
Psychiatry	41 (4.1)	456 (6.5)	
Emergency medicine	21 (2.1)	610 (8.7)	
Family medicine	66 (6.6)	539 (7.67)	
Others	331 (33.1)	1773 (25.3)	
Marital (coupled)	187 (18.6)	2709 (38.6)	< 0.001
History of depression	351 (34.9)	3324 (47.3)	< 0.001

Number (%): frequency and percentage.

*n* total number, *SD* standard deviation, *P* value by t-test and chi-square test.

#### Within-residency assessments

Participants were contacted via e-mail at months 3, 6, 9, and 12 of their first year of residency and asked to complete the PHQ-9. They were also queried regarding their rotation setting, perceived medical errors, work hours, and sleep during the past week and the occurrence of a series of non-residency life stressors (serious illness; death or serious illness in a close family member or friend, financial problems; end of a serious relationship; or becoming a victim of crime or domestic violence) during the past 3 months. Chinese residents were also asked three questions about workplace violence, both at baseline and at each of the quarterly assessments: Have you experienced, observed or do you fear being physically assaulted, verbally abused, or threatened by a patient or their relatives?

### Statistical analyses

All analyses were performed using SAS version 9.4. Statistical tests were 2-sided with a significance threshold of *P* < 0.05.

#### Prevalence of depressive symptoms

To investigate whether there was a significant change in depressive symptoms or in depression prevalence during the first year of residency, we compared baseline PHQ-9 depressive symptoms and depressive symptoms at the 3-, 6-, 9-, and 12-month assessments through a series of paired t-tests. We also compared the percentage of subjects meeting diagnostic criteria for depression between baseline and during residency through a series of McNemar’s tests.

#### Predictors of depression during residency

To identify baseline variables that predict change in depressive symptoms during residency, we used Pearson correlations for continuous measures and Chi-square analyses for nominal measures. Significant variables were subsequently entered into a stepwise linear regression model to identify significant predictors while accounting for collinearity among variables. We also assessed the association between within-residency variables, assessed through quarterly surveys (work hours, occurrence of medical errors, hours of sleep, non-residency stressful life events and, in China only, fear of workplace violence and stressful events) and change in depressive symptoms during residency. Specifically, through a series of generalized estimating equation (GEE) analyses to account for correlated repeated measures within subjects, within-internship factors were incorporated as predictor variables, and associated baseline factors were used as covariates.

### Ethical approval

This study design and informed consent procedure was approved by the Ethics committees of Peking Union Medical College, the University of Michigan, and Shanghai Jiao Tong University (No. ML16041).

### Previous presentations

A slightly modified draft has been deposited on Med Archives: https://www.medrxiv.org/content/10.1101/2020.04.12.20049882v1.

## Results

In China, 1006 (60%) subjects completed at least one follow-up survey and provided gender and age and were included in the analysis. In the US, 7028 (85%) subjects completed at least one follow-up survey and were included in the analysis.

### Prevalence of depressive symptoms

The mean PHQ-9 score in China was significantly higher than the US score at baseline (mean diff = 1.37, 95% CI 1.11–1.62; *P* < 0.001), and at 3 (mean diff = 0.48, 95% CI 0.11–0.84; *P* = 0.01), 6 (mean diff = 0.56, 95% CI 0.14–0.99; *P* = 0.009), 9 (mean diff = 0.66, 95% CI 0.23–1.09; *P* = 0.003) and 12 months (mean diff = 0.96, 95% CI 0.46–1.47; *P* < 0.001) of residency. Among first year residents in China, the mean HQ-9 depressive symptom score increased significantly from baseline (3.99 ± 3.97) to 3 months (6.21 ± 4.90; *P* < 0.001), 6 months (6.53 ± 5.18; *P* < 0.001), 9 months (6.57 ± 5.09; *P* < 0.001), and 12 months (6.76 ± 5.56; *P* < 0.001) of residency. In the US sample, PHQ-9 scores also increased significantly from baseline (2.62 ± 3.02) to 3 months (5.73 ± 4.34; *P* < 0.001), 6 months (5.96 ± 4.55; *P* < 0.001), 9 months (5.91 ± 4.62; *P* < 0.001), and 12 months (5.80 ± 4.68; *P* < 0.001). There was no significant difference in PHQ-9 change between the China and US cohorts.

**Figure 1:**
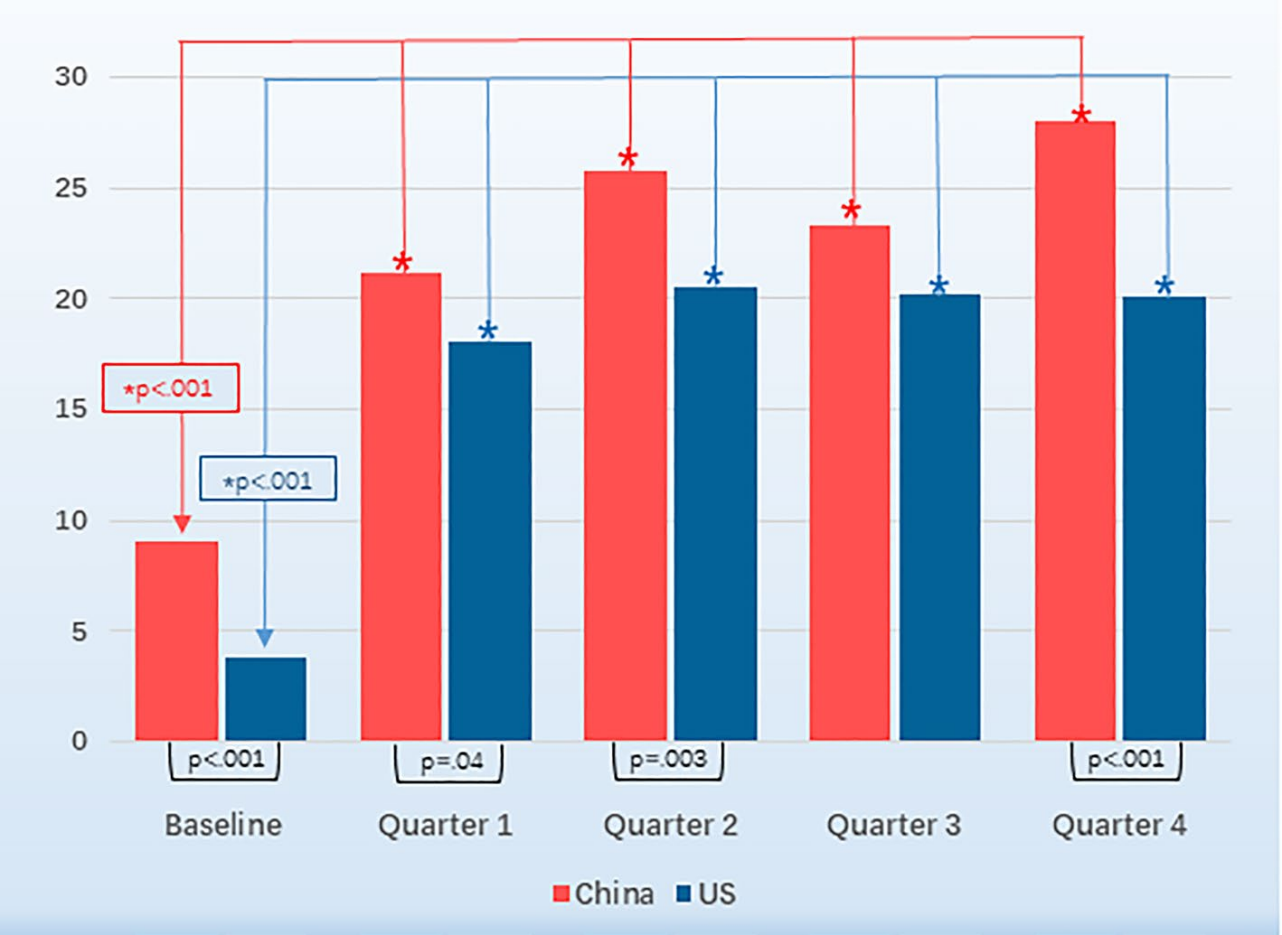
Depression Rates of Residents in China (red) and the United States (blue). The PHQ depression rates of residents in each quarter is significantly higher than the baseline in both countries.

Among residents in China, the percentage of subjects meeting diagnostic criteria for depression increased from 9.1% (91/1006) at baseline to 21.1% (162/767), 25.7% (161/626), 23.4% (138/591), and 28.0% (139/497) at the 3-, 6-, 9-, and 12-month points of residency, respectively (Fig. 1). Overall, 35.1% (353/1006) of subjects in China met the criteria for major depression at least once during the first year of residency. In the US sample, the percentage of subjects meeting PHQ depression criteria increased from 3.9% (271/7028) at baseline to 18.1% (1143/6314), 20.6% (1146/5572), 20.2% (1054/5232), and 20.1% (965/4803) at the 3-, 6-, 9-, and 12-month points of internship, respectively (Fig. 1). In all, 34.9% (2454/7028) of US subjects met the criteria for major depression at least once during internship. There was no significant difference in the proportion of residents meeting criteria for depression between China and the US (35.1 vs. 34.9%, OR = 0.99, 95% CI 0.86–1.14; *p* = 0.91).

### Baseline predictors of depression

Among nine baseline variables tested in both Chinese and US residents, three were significant predictors of PHQ-9 score change within residency in both China and the US: personal history of depression, stressful life events and a lower PHQ-9 baseline depression symptom level (Table 2). Young age was a predictor of depressive symptoms only in China, while female gender, neuroticism score, early family environment and not being coupled were predictors of depression only in the US (Table 2). Fear of workplace violence was also a significant predictor in China.

**Table 2:** Baseline predictors of change in PHQ-9 score during residency (average across all 4 follow-ups) in China and the US.

	China		US	
	Pearson *r*	*p* value	Pearson *r*	*p* value
Age	**− 0.17**	**< 0.001**	**− **0.001	0.92
Neuroticism	**− **0.04	0.26	**0.14**	**< 0.001**
Early family environment	**− **0.009	0.78	**0.09**	**< 0.001**
Baseline depressive symptoms	**− 0.39**	**< 0.001**	**− 0.28**	**< 0.001**
Gender (female)	0.04	0.29	**0.08**	**< 0.001**
Marital status (coupled)	0.07	0.08	**0.05**	**< 0.001**
Personal history of depression	**0.23**	**< 0.001**	**0.26**	**< 0.001**
Stressful life events	**0.11**	**0.007**	**0.07**	**< 0.001**
Specialty	0.08	0.76	0.02	0.88
Fear of workplace violence	**0.24**	**< 0.001**	NA	NA

Significant correlations are bolded. *NA* not available. All significant factors in the model are bolded.

### Within-residency predictors of depression

Three within-residency factors were significantly associated with an increase in depressive symptoms in both countries: work hours, non-internship stressful life events and average sleep hours (Table 3). Reported medical errors were significantly associated only in the US. In China, 13.3% (80/600) of residents reported experiencing violence by patients or patients’ relatives, while 39.3% (236/600) reported witnessing violence and 28.3% (170/600) reported fear of violence. While almost 20% of US residents reported errors each quarter, less than 2% of Chinese residents reported errors.

**Table 3:** GEE model of significant baseline and within residency predictors in China and the US.

	China		US	
	*ß*	*p* value	*ß*	*p* value
Age	**− 0.28**	**< 0.001**		
Gender (female)			0.08	0.2924
Marital status (coupled)			**− 0.24**	**< 0.001**
Neuroticism			**0.12**	**< 0.001**
Early family environment			**0.03**	**< 0.001**
Baseline depressive symptoms	**0.49**	**< 0.001**	**0.41**	**< 0.001**
Personal history of depression	**1.71**	**< 0.001**	**0.90**	**< 0.001**
**Residency factors**				
Work hours	**0.03**	**< 0.001**	**0.04**	**< 0.001**
Sleep hours	**− 0.34**	**< 0.001**	**− 0.21**	**< 0.001**
Medical errors	0.17	0.13	**0.87**	**< 0.001**
Stressful life events	**0.70**	**< 0.001**	**0.34**	**< 0.001**
Fear of workplace violence	**0.20**	**< 0.001**		

Empty space: these factors were not originally significant or not measured. All significant factors in the model are bolded.

## Discussion

This study is the first prospective large-scale assessment of depression during the first year of residency training in China. Despite cultural and structural differences, we find striking parallels in prevalence and system predictors of depression between China and the US, with about 35% of participants fulfilling criteria for depression at least once during the first year of residency in both systems. In addition, most baseline and within-residency predictors were common between countries.

Importantly, we identified several individual baseline factors associated with the development of depressive symptoms during residency. Consistent with previous studies^9^, a history of major depression and of other stressful life events, as well as reduction of sleep^24^, longer work hours^10,25^ and stressful events outside of residency^10^ were robust predictors of the observed increase in depression during residency in both countries. Notably, residency specialty was not associated with the development of depression in either country.

Interesting differences in the prevalence and risk factors for depression were also identified between China and the US. While the higher baseline score may be attributed to the significantly shorter transition time between medical school and the start of residency in China (two weeks) in contrast to the US (two months), the higher scores throughout the year suggest that young Chinese physicians may face additional stressors. For example, violence by patient or patients’ relatives against providers has recently been much discussed^5,26^, and fear of such violence was a significant predictor in the final model of depression during residency in China. Younger age was also a significant predictor of depression in China but not in the older US sample. This could be due to the earlier starting time of residency in China (after five years of a Bachelor in Medicine) than in the US (four years of undergraduate studies followed by four years of medical school). The difference in reporting of medical errors (20% in the US vs. 2% in China) is likely a consequence of more supervision, such that these younger first year residents in China would not feel being directly responsible for a medical error.

The personality trait, neuroticism, is the strongest and among the best replicated predictors of depression during residency in the US^10,27^. In contrast, neuroticism has no predictive power for depression for residents in China. The non-significant trend of the neuroticism-depression association was in the opposite direction. This finding supports previous work suggesting that anxiety-related personality traits serve as vulnerability factors for disease in individualistic cultures, such as the US, but serve as protective factors in collectivist cultures such as Japan and China^28,29^, but is different from the findings of a large meta-analysis of depression in East-Asians which found high correlation between neuroticism and depression^30^.

Our study has a number of limitations. First, since only about half of residents completed our questionnaires, it is conceivable that those who are suffering of depression symptoms during residency are more motivated to complete assessments. Second, we were only able to include residents from large hospitals in Shanghai and Beijing, who likely are not representative of training physicians across China. Finally, we focused on first-year residents in this study. Prevalence and predictors of depression may be different in more advanced training physicians.

In summary, this study is the first large-scale to assess depression during the first year of residency training in China. Based on large sample sizes from China and the US, the data consistently showed that the first year of residency is a stressful time leading to a marked increase of depression, with about 35% of participants fulfilling criteria for depression at least once during the first year of residency in both countries. The finding that individual risk and protective factors differ between the two countries highlights the importance of developing tailored interventions to improve resident mental health based on sociocultural context. At the same time, the identification of high workload and lack of sleep as major modifiable factors that could improve trainee mental health in both countries suggest that successful interventions developed in one culture may succeed in the other.

Although depression rates among training physicians in the US remains high, the average increase in depressive symptoms associated with internship has decreased in recent (pre-pandemic) years^31^. Decreased work hours and increased utilization of mental health services were important drivers of the improvement, suggesting that targeting these factors are promising avenues to further improving the mental health of training physicians in both the US and China.

## Data Availability

The US datasets generated and/or analyzed during the current study are a subset of the Intern Health Study and are available in the open ICPSR repository, https://www.openicpsr.org/. The Chinese datasets used and/or analyzed during the current study are available from corresponding author Dr. Li on reasonable request.
